# The Impact of Stigma on Treatment Services for People With Substance Use Disorders During the COVID-19 Pandemic—Perspectives of NECPAM Members

**DOI:** 10.3389/fpsyt.2021.634515

**Published:** 2021-03-02

**Authors:** Lisa Dannatt, Ramdas Ransing, Tanya Calvey, Florian Scheibein, Noha Ahmed Saad, Tomohiro Shirasaka, Rodrigo Ramalho, Sagun Pant, Ramyadarshni Vadivel, Kristiana Siste, M. J. Stowe, Kamal Narayan Kalita, Saïd Boujraf, Roberta Testa, Sidharth Arya, Nirvana Morgan, Paolo Grandinetti

**Affiliations:** ^1^Department of Psychiatry and Mental Health, University of Cape Town, Cape Town, South Africa; ^2^Department of Psychiatry, Bhaktshreshtha Kamalakarpant Laxman (BKL) Walalwalkar Rural Medical College, Pune, India; ^3^Faculty of Health Sciences, School of Anatomical Sciences, University of the Witwatersrand, Johannesburg, South Africa; ^4^School of Health Science, Waterford Institute of Technology, Waterford, Ireland; ^5^State Drug Dependence Treatment Centre, Ain Shams University, Cairo, Egypt; ^6^Department of Psychiatry, Teine Keijinkai Medical Center, Sapporo, Japan; ^7^Department of Social and Community Health, School of Population Health, University of Auckland, Auckland, New Zealand; ^8^Department of Psychiatry, Institute of Medicine, Tribhuvan University, Kirtipur, Nepal; ^9^Department of Mental Health and Addictions, Waikato District Health Board, Hamilton, New Zealand; ^10^Department of Psychiatry, Medical Faculty, Universitas Indonesia-Cipto Mangunkusumo Hospital, Jakarta, Indonesia; ^11^Department of Family Medicine, Faculty of Health Sciences, School of Medicine, University of Pretoria, Pretoria, South Africa; ^12^Department of Psychiatry, Lokopriya Gopinath Bordoloi Regional Institute of Mental Health (LGBRIMH), Tezpur, India; ^13^Faculty of Medicine and Pharmacy, Sidi Mohamed Ben Abdellah University, Fez, Morocco; ^14^Department of Mental Health, Azienda Sanitaria Locale (ASL) 1 Avezzano-L'Aquila-Sulmona, L'Aquila, Italy; ^15^State Drug Dependence Treatment Centre, Institute of Mental Health, Pt. Bhagwat Dayal Sharma (BDS) University of Health Sciences, Rohtak, India; ^16^Faculty of Health Sciences, University of the Witwatersrand, Johannesburg, South Africa; ^17^Department of Mental Helth, Azienda Sanitaria Locale (ASL) Teramo, Teramo, Italy

**Keywords:** stigma, COVID-19, substance use disorder, access to treatment, pandemic, mental health

## Introduction

Stigma is a mark of shame, disgrace, or disapproval which results in an individual being rejected, discriminated against, and excluded from society (1). Stigma toward individuals with substance use disorders (SUDs) affects the emotional, mental, and physical health of individuals (2). People with SUD are often viewed as unpredictable, dangerous, and morally responsible for their condition (2). These prejudiced and discriminatory views of the community may lead to reduced access to care, inability to make decisions regarding treatment, and forced or coerced treatment (2). Further, stigma negatively affects the policies and programs intended for the management of substance use and other addictive disorders (2). Moreover, people with addictive disorders may develop self-stigma influencing their behavior, including decreased use of healthcare services with consequent poorer health outcomes (3). Internalized stigma and self-stigma have been linked to increases in psychological distress and poorer quality of life (4, 5). People with substance use disorders (SUDs), in particular, may face significant stigmatization by healthcare practitioners (6). Of significant concern during the COVID-19 pandemic is that people with addictive disorders and concurrent COVID-19 may not be provided with adequate care (7). Therefore, people with SUDs may be experiencing increased stigmatization in different countries during the COVID-19 pandemic. This exacerbated stigma and discrimination toward people with SUDs may lead to inadequate care or poor attention from clinicians, policymakers, and other stakeholders.

To explore this important issue, in March 2020, members of the Network of Early Career Professionals working in the area of Addiction Medicine (NECPAM) were asked to share their experiences, observations, relevant published literature, and opinions from their respective countries. NECPAM members are frontline health care workers, e.g., doctors, psychiatrists, and employees of non-governmental organizations, who are actively involved in the care of people with SUDs. Opinions from NECPAM members were also requested via a qualitative online survey, of which 28 responded. Responses from the online survey were grouped into themes. Of 28 respondents there were 14 NECPAM members (six female and eight males) hailing from all WHO regions who stated that stigma in some form had affected addiction care during the COVID 19 pandemic. The opinions of these members representing 10 countries (Italy, India, Nepal, Morocco, South Africa, Egypt, Ireland, Indonesia, Japan, and New Zealand) are represented in this opinion piece. Here, we discuss the impact of stigma on individuals with substance use and other addictive disorders during COVID-19 in three themes: (i) policy, (ii) access to adequate services, and (iii) marginalized populations.

## Subsections Relevant for the Subject

### Policy

Members felt that during the COVID-19 pandemic SUDs and behavioral addictions had not featured significantly in policy and program planning in most settings. Stigma toward individuals with substance use and other addictive disorders was thought to be one of the causes as these individuals may be seen as less deserving of care. This is evidenced by the quote below from a psychiatrist in South Africa:

“*Overall, I think stigma toward people who use drugs has played a significant role in service planning and execution with the sense that these clients may not deserve or warrant the care and attention that people with medical disorders do. This has felt like a worsening of the usual stigma toward people who use alcohol and other drugs.”*

When hospital-based services were planned and restructured, mental health and addiction wards in some settings had been repurposed into COVID-19 wards, with little future planning by policymakers regarding addiction services. An example given from Morocco was that, before the COVID-19 pandemic, the Ministry of Health had launched the National Strategic Plan of Prevention and Care of Addictive Disorders in January 2018 (8). This program addressed several aspects of the stigmatization of people with SUDs including their rights to access healthcare and to preserve their dignity (8). However, during the COVID-19 pandemic, there was no official plan to manage the support and treatment of substance use and other addictive disorders. The addiction health services were reduced to a minimum. Addictions input was provided by continued general psychiatric services. Addiction centers were also used to treat COVID-19 positive mental health care users. Substance users were then offered care when required in psychiatric departments of hospitals (9). Similar experiences were reported by NECPAM members from South Africa, Nepal, and Japan (10). Conversely, a member from New Zealand reported that although the initial health planning and policies were centric to the pandemic itself; there was an early response to address an increase in SUD and other addictive disorders during the COVID-19 pandemic and lockdown. An initial survey aimed at both service providers and people with SUDs identified the potential negative consequences due to harmful substance use during the COVID-19 pandemic (11). These included hesitance in seeking professional help, with most individuals with SUD opting for not reaching out for support (11). Moving with the demand, the New Zealand government mobilized new funding toward fast-tracking mental health services (12). Emphasis has been made on promoting knowledge about substance use and gambling harms, reducing stigma, and facilitating enhanced access to support.

### Access to Services

According to our members, stigma affects access to services for people with substance use and behavioral addictions in a variety of ways and our members reported several different examples of this. Members from Nepal, South Africa, and New Zealand perceived this stigma as particularly prevalent toward people who accessed opioid use treatment services and other harm reduction-related services. The stigma toward people who use opioids and how this serves as a barrier to effective treatment for opioid use disorders is well-described in the literature prior to the COVID-19 pandemic (13). Regarding these specific countries, there is evidence from the literature prior to the COVID-19 pandemic that stigma toward opioid users is a barrier to access to treatment (14–16). However, establishing a Healthline helpline to facilitate testing and treatment seems to have reduced the stigma around access for service users in New Zealand (17). Some members reported their personal observation and experience. A member from Egypt described that social stigma leads to inequality in accessing medical services as communities hold individuals with SUDs morally responsible for their illness and, in her opinion, this may lead to denial of access to treatment. Members from India reported that patients presenting to treatment services were frequently questioned and fined as police believed them to be in breach of the local COVID-19 lockdown policies. Additionally, it was perceived by members from India that people who present with psychotic disorders related to substance use are more severely stigmatized than people with psychosis who do not use drugs. A member from Indonesia reported that individuals with substance use and behavioral addiction disorders are often faced with restrictions of access to healthcare services. Although some protocols were developed for people with SUD during the COVID-19 pandemic, the member noted that no policies were made to coordinate services for people with behavioral addictions. The absence of specific protocols for people with behavioral addictions during the pandemic was also noted by several other members. Therefore, the Indonesian government has released a specific protocol of HIV-AIDS health services during COVID-19 and the Indonesian Psychiatric Association in tandem published practice guidance for a psychiatrist in COVID-19 healthcare centers which also manages patients with addiction disorders. Regrettably, no policies have been made to coordinate services for patients with behavioral addiction during the COVID-19 pandemic. A member from Japan discussed that patients accessing addiction services who presented with fever were refused transportation to emergency centers or refused hospitalization Therefore, to solve these problems, the Disaster Support Committee of the Japanese Society of Psychiatry and Neurology has created guidelines on how to build and respond to medical systems for psychiatric patients with infectious diseases (18).

### Street Based People and Special Population Groups

A member from South Africa stated that stigma toward street-based people contributed to inadequate service planning. In South Africa, during the COVID-19 policy of lock-down mass temporary shelters were created for street-based people in various cities (19). Although people in shelters were provided with essentials, there was inadequate planning for people with SUD's. The NECPAM member from South Africa observed that some street-based people who were moved to shelters suffered uncomfortable and unsupported withdrawal symptoms. The Department of family medicine at the University of Pretoria stepped in with the Community Orientated Substance Use Program (COSUP) to provide an emergency substance use management response within the shelter in the City of Tshwane (19). In Ireland, a range of COVID-19 policies were enacted which focused on people experiencing homelessness and using drugs (20). These included providing single-room occupancy housing for COVID-19 high-risk populations; a reduction in the homeless service occupancy levels to increase safety; dropping induction times for opioid agonist maintenance treatment (OAMT) from 12–14 weeks to 2–3 days; greater availability of takeaway doses of OAMT; delivering OAMT to those isolating; increasing availability of naloxone to all people with OAMT prescriptions and increasing availability of benzodiazepine maintenance treatment which was directly delivered to accommodation services (20). Some countries had formulated measures to prevent stigma toward marginalized populations during the COVID-19 pandemic. In Japan, the Ministry of Justice established a consultation desk for human rights issues (21). These consultations were provided telephonically or via the internet (21). In New Zealand, with the initiation of a nationwide lockdown, services were mobilized to enable emergency placement of vulnerable individuals in line with physical distancing measures (22). Services have been delivered through digital platforms, albeit with difficulty, recognizing the need to increase such technological means (11, 13).

## Discussion

Stigma toward people with SUDs could be one of the possible reasons for non-prioritizing SUDs and addiction services during the COVID-19 pandemic. However, people with SUD's are a vulnerable population and non-consideration can lead to serious consequences, such as increased mortality, including death by suicide. Multi-level interventions targeting multiple stages are required to address these complex issues at the policy, community, and individual level. Reframing the policy or guidelines to create a balance between COVID-19 pandemic services and addiction services is needed to provide affordable, safe, accessible, and effective care for people with SUDs. Moreover, we would like to recommend some suggestions to reduce the stigma toward people with SUDs and improve access to care during the pandemic.

Moreover, we would like to recommend some suggestions, that should be emphasized during the Pandemic, to reduce the stigma toward people with SUDs. First, the Involvement of policymakers; health care providers; and other key stakeholders for planning and co-ordinations for healthcare service provision and adjust according to a known or perceived demand. Second, Addiction services should be integrated with other health services and decentralized to provide patients with accessible health care. Third, Mass media campaigns on television or the internet should be conducted to reduce stigmatization in the community for people with SUDs. Fourth, the physical and mental health conditions of patients with SUDs should be acknowledged and addressed as a priority. Fifth, recognize the role of caregivers or relatives of people with SUDs.

## Author Contributions

LD wrote the first draft of the paper. RR, NM, and PG revised and all authors reviewed and commented and approved the final draft. All authors participated in on-line forums and contributed their opinions.

## Conflict of Interest

The authors declare that the research was conducted in the absence of any commercial or financial relationships that could be construed as a potential conflict of interest.
